# Regulation of Pro-Inflammatory Macrophage Polarization via Lipid Nanoparticles Mediated Delivery of Anti-Prostaglandin-E2 siRNA

**DOI:** 10.3390/cimb45010001

**Published:** 2022-12-20

**Authors:** Ahmad Almatroudi, Mohammed A. Alsahli, Mansoor Ali Syed, Amjad Ali Khan, Arshad Husain Rahmani

**Affiliations:** 1 Department of Medical Laboratories, College of Applied Medical Sciences, Qassim University, Buraydah 52571, Saudi Arabia; 2 Translational Research Lab, Department of Biotechnology, Faculty of Natural Sciences, Jamia Millia Islamia, New Delhi 110025, India; 3 Department of Basic Health Science, College of Applied Medical Sciences, Qassim University, Buraydah 52571, Saudi Arabia

**Keywords:** inflammation, macrophage polarization, lipid nano particles, prostaglandin E2, siRNA

## Abstract

Pro-inflammatory macrophage polarization is crucial in acute inflammatory diseases like Acute lung injury (ALI), and acute respiratory distress syndrome (ARDS). Prostaglandin E2 (PGE2) is believed to promote inflammation in such cases. Therefore, our study aimed to deliver anti-prostaglandin E synthase 2 small interfering RNA antibodies (anti-PGE2-siRNA) through lipid nanoparticles (LNPs) in RAW264.7 (The murine macrophage cell line) to find a possible cure to the acute inflammatory diseases. LNPs were synthesized by using thin layer evaporation method and were characterized by dynamic light scattering (DLS), Zeta potential, SEM and TEM analysis. The obtained NPs were spherical with an average size of 73 nm and zeta potential +29mV. MTT assay revealed that these NPs were non-toxic in nature. Gel retardation assay displayed 5:2 ratio of siRNA and NPs as the best siRNA:LNPs ratio for the delivery of siRNA into cells. After siRNA delivery by using LNPs, real time gene expression analysis revealed significant decrease in the expression of PGE2. Western blot results confirmed that silencing of PGE2 gene influence inducible nitric oxide synthase (iNOS) and interlukin-1β (1L-1β), markers involved in pro-inflammatory macrophage polarization. Our study revealed that LNPs synthesized in present study can be one of the effective methods to deliver anti-PGE2-siRNA to control pro-inflammatory macrophage polarization for the treatment of acute inflammatory response.

## 1. Introduction

Macrophages are highly plastic in nature and thus they can be polarized into several subsets under different stimuli. Bacterial lipopolysaccharide (LPS) can stimulate macrophages to their inflammatory state while IL-4 can stimulate them into anti-inflammatory phenotype [1,2]. A highly complex set of regulatory network governs this macrophage polarization [3]. Proinflammatory macrophages generally kill pathogens and present their antigens to the adaptive immune system [4]. Subsequently, the anti-inflammatory cells resolve inflammation and repair the damage [5]. The balance of the two phenotypes is crucial during inflammation or injury. In acute respiratory distress syndrome (ARDS), acute lung injury (ALI), and during foreign body response (FBR) this balance is somewhat disrupted [6,7]. Continuous M1 polarization can release excessive proinflammatory cytokines like interleukin-1 (IL-1), nitric oxide (NO), tumor necrosis factor-α (TNF-α), and reactive oxygen species (ROS) to induce a severe inflammatory response [6,8]. Prostaglandin E2 (PGE2) significantly influences the progression of the inflammatory response by macrophages [9]. Their production is markedly increased in inflamed tissues, and they help to produce the key symptoms of severe inflammation [10]. Recently, PGE2 was reported to augment M1 polarization. Suppression of PGE2 also promoted M2 macrophage polarization and decreased the allergic airway inflammatory cell infiltration in Abx-treated mice [11]. Therefore, PGE2 can be a potential therapeutic target to attenuate M1 polarization. Some new strategies are in progress to down-regulate the expression of PGE2 and prevent M1-induced severe inflammation [12]. 

Small-interfering RNAs (siRNAs), also known as short interfering RNA or silencing RNA, are a type of double-stranded non-coding RNAs that functions through the RNA interference (RNAi) pathway. They are usually 20–24 base pairs (commonly 21 bp) long, making them similar to miRNA [13]. They have recently been demonstrated to induce transcriptional gene silencing in humans [14]. An RNA-induced silencing complex (RISC) system (an endogenous enzyme system), initiates gene silencing with siRNA. In theory, specific siRNA can target and silence any gene. Thus, it can be used to down-regulate the expression of PGE2. However, several significant obstacles prevent siRNA from being delivered for therapeutic applications. First, in biological fluids, "naked" siRNAs are unstable and easily are destroyed by nucleases, resulting in their poor accumulation at target sites [15]. Second, because of its large size, (13 to 15 kDa), and negative charges, siRNA cannot cross the cell membrane [16]. Moreover, systemically injected siRNAs can accumulate in the liver and kidney. The kidneys remove siRNA from circulation and excrete them [17]. To get over these aforementioned obstacles, suitable delivery vehicles are consequently required. Lipid-based nanoparticles (LNPs) such as liposomes are frequently used for nucleic acid delivery within target cells. The LNPs are biocompatible, biodegradable, less toxic, structurally flexible, and can be easily produced on a large scale [18,19,20]. Therefore, LNPs can be an ideal vehicle for the delivery of siRNAs.

This work was designed to combat acute inflammation and control the proinflammatory M1 polarization by silencing PGE2 through LNP-mediated Si-RNA delivery.

## 2. Materials and Methods

### 2.1. Chemicals and Reagents

RAW264.7 (The murine macrophage cell line) was procured from NCCS, Pune, India. Dulbecco’s Modified Eagle Medium (DMEM) (Gibco, Grand Island, NY, USA), FBS (heat inactivated) (Gibco, Grand Island, NY, USA) and 1% antibiotic-antimycotic (Gibco, Grand Island, NY, USA) were purchased. Trizol reagent (Ambion, Elk Grove, CA, USA, cDNA synthesis kit (verso, cDNA synthesis kit, Thermo Scientific, Waltham, MA, USA), SYBR green (PowerUp™ SYBR™ Green Master Mix, Applied Biosystems, Thermo, Waltham, MA, USA) were purchased. All routines chemicals were purchased from local supplier.

### 2.2. Synthesis of Nanoparticles

Synthesis of lipid nanoparticles (LNPs) is done by a thin layer evaporation method [21]. In brief, DSPC, cholesterol, and polyethylenimine (PEI) (4:1:2) were dissolved in Chloroform and Methanol (1:1) and kept on magnetic stirrer for 4 hr at 25 °C and 200 rpm. Next, the solvent was evaporated using a rotary evaporator (DLAB RE-100 Pro) at 50 °C, 40 RPM, to obtain a thin layer. The obtained thin layer of lipids was dispersed in doubled distilled water by sonication (Sonics, Vibra cell VCX 500) at 50 amplitude (10 s on-off cycle) for 10 min and extruded using a 200 nm then 100 nm polycarbonate membrane at 50 °C to control the LNPs size.

### 2.3. Characterization of LNPs

Nano Zetasizer system (Malvern Instruments) was used for dynamic light scattering (DLS) analysis at 25 °C, 0.8872 mPas medium viscosity, and 1.59 medium refractive index for the evaluation of hydrodynamic size distribution of the particles, zeta potential and disparities in the colloidal sample. The analysis was done after loading the material into a quartz microcuvette and measurements was done accordingly.

Transmission electron microscopy (TEM-Philips, EM-410LS, JEOL, Osaka, Japan) and Scanning electron microscopy were used to examine the morphology of LNPs (SEM). For TEM analysis, a little drop of the highly diluted sample was evenly distributed throughout the copper grid and dried at room temperature. Similarly, samples were prepared for SEM analysis followed by gold coating in a sputter coater to examine the samples. Micrographs were captured on SEM (Nova NanoSEM 450, at accelerating voltage of 5 keV.

### 2.4. Cell Culture and Maintenance

RAW264.7 (murine macrophage cell line) was procured from NCCS, Pune, India, and maintained in Dulbecco’s Modified Eagle Medium (DMEM) (Gibco, Grand Island, NY, USA) supplemented with 10% FBS (heat inactivated) (Gibco, Grand Island, NY, USA) and 1% antibiotic-antimycotic (Gibco, Grand Island, NY, USA). Cells were incubated at 37 °C in a humidified incubator saturated with 5% CO_2_. These macrophages were stimulated by LPS treatment (100 ng/mL). 

### 2.5. Biocompatibility of the LNPs

Biocompatibility was assessed using MTT assay [22,23]. Briefly, 7 ⤫ 10^3^ cells (RAW264.7) in their exponential growth phase were seeded in a 96-well plate and kept in an CO_2_ incubator at 37 °C for 24 h. Different concentrations of LNPs were used to treat the cells and after 24 h, 20 μL MTT reagent was added in each well and incubated for 4 h. Formazan crystals were dissolved by adding 150 μL of DMSO in each well. Absorbance was measured using a microplate reader at 590 nm (imark BIO-RAD microplate reader). The percentage viability of cells was calculated using following equation:

% Viability = 100 − [(Absorbance of control − Absorbance of treated)/Absorbance of control] * 100

### 2.6. Binding Efficiency of siRNA with LNPs through Gel Retardation Assay

Different quantities of LNPs (1–7 µg) were made to react with a fixed amount of siRNA (10 µg) for 5 min at room temperature. The mixture was run on the denaturing agarose gel (1.5 %) at 100 V for 20 min to find the best combination ratio of siRNA:LNP.

### 2.7. RNA Extraction and Real-Time Quantitative PCR

Realtime PCR was performed to check the mRNA expression of target genes. Following the manufacturer’s instructions, RNA was extracted from the siRNA treated RAW264.7 cell line using Trizol reagent (Ambion, Elk Grove, CA, USA). Then, using a cDNA synthesis kit (verso, cDNA synthesis kit, Thermo Scientific, Waltham, MA, USA) cDNA was synthesized. To perform qPCR, cDNA was diluted 1:20 times. With the use of SYBR green (PowerUp™ SYBR™ Green Master Mix, Applied Biosystems, Thermo, Waltham, MA, USA), the mRNA levels were assessed. The Real-time PCR System QuantStudio 6 Flex (Applied Biosystems, Waltham, MA, USA) was used for the experiment, and the thermocycling settings were as follows: initial denaturation 95 °C for 5 min, denaturation 95 °C for 30 s, annealing (54 °C) for 35 s, and extension 72 °C for 40 s. Data was analyzed by the Livak (ΔΔCt) method [23]. Following primers are used to detect the expression of target genes by qRT-PCR analysis: PGE2: Forward-5′ GAA GGA CTG AGA TCA AAT TCT C 3′, Reverse-5′ ATG ACA GAG GAG TCA TTG AG 3′, Β-ACTIN: Forward- 5′ TGA CCC AGA TCA TGT TTG AG 3′, Reverse- 5′ ATC CCA TCA CAA TGC CTG 3′, iNOS: Forward- 5′ TCC TGG AGG AAG TGG GCC GAA G 3′, Reverse- 5′ CCT CCA CGG GCC CGG TAC TC 3′,IL-1β: Forward- 5′ TCA GGC AGG CAG TAT CAC TC 3′, Reverse- 5′ CAT GAG TCA CAG AGG ATG GG 3′.

### 2.8. Protein Extraction and Western Blot

RIPA lysis buffer (Thermo scientific, USA) supplemented with protease and phosphatase inhibitor (Thermo scientific, USA) was used to extract the protein. A Bio-Rad kit was used to quantify the extracted protein and was quantified by Bradford assay kit (Bio-Rad, Hercules, CA, USA). SDS-PAGE was used to resolve the protein, which was subsequently transferred onto PVDF membrane. After the membrane had been blocked with 5% skimmed milk, the primary mouse monoclonal antibody, Anti-iNOS (BioLegend, USA) and Anti-IL-1β antibodies (sc-12742, Santa Cruz Biotechnology, Inc., Santa Cruz, CA, USA) were added onto the membrane for an overnight incubation at 4 °C. It was followed by an incubation with HRP-conjugated secondary antibody at room temperature for 1 h. Thereafter, protein bands were visualized using Chemi-Doc XRS (Bio-Rad) by ECL substrate (Bio-Rad).

### 2.9. Statistical Analysis

All the experiments were run in triplicate. Data were expressed as the mean ± SEM. GraphPad Prism 3.0 software (San Diego, CA, USA) was used for the statistical analyses. One-way ANOVA and Dunnett tests were further used. Values of *p* < 0.05 were considered statistically significant

## 3. Results

### 3.1. Synthesis and Characterization of LNPs

**Figure 1:**
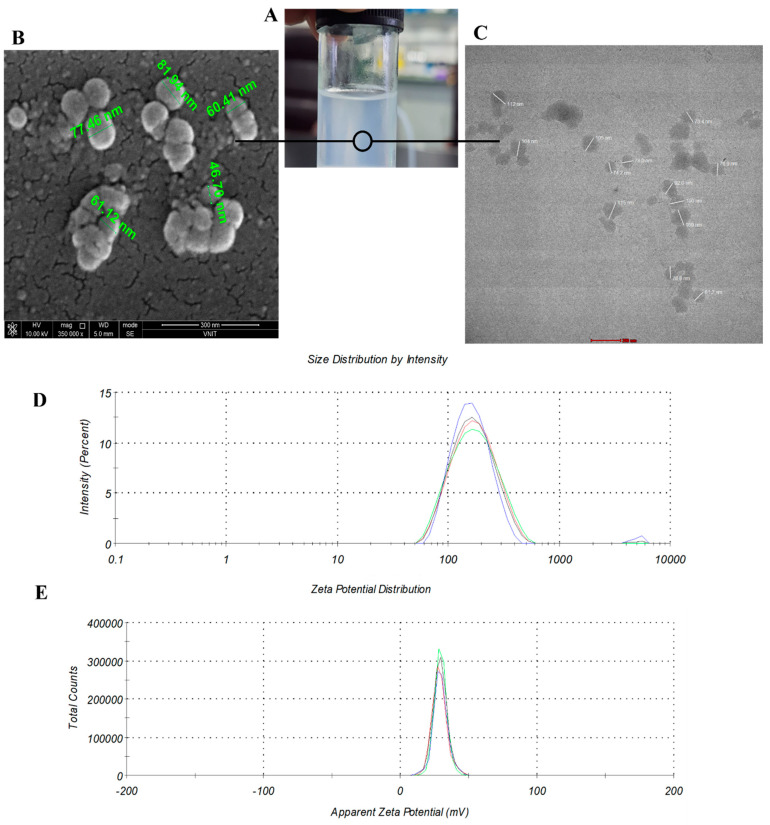
Physical characterization of lipid nanoparticles. (**A**) Appearance of lipid nanoparticles (LNPs) solution, (**B**) SEM analysis of LNPs (**C**) TEM analysis of LNPs (**D**) Size distribution by DLS and, (**E**) Zeta potential.

LNPs were successfully synthesized using the above-mentioned protocol. Movement of the particle is random and translational. Surface charge on the NPs causes adsorption of solvent (creating hydration layer), forming corona and make it more complex thus causing altered surface of the particle. The scattered light is not only by the NPs but it is a constructive particle (NP and hydration layer) that scatter the light. DLS determine the hydrodynamic size of the particle which is the hypothetical measurement and not the ac-tual size of the synthesized NP. Therefore, DLS provide indicative size [24,25]. In the present study, DLS analysis revealed the hydrodynamic size of 154 nm [Figure 1].

DLS data is used to calculate PDI, typically depicts the intensity of light scattered by various fractions of the particles differing in their size. Our DLS data showed PDI (Poly-dispersity index) as 0.163 and this indicates that particles are considerably mono-disperse in nature not in aggregating form. More PDI value mean more heterogenous NPs are there. DLS has very low resolution which is the limitation of the technique [26]. For example, DLS is unable to distinguish between particles of 90 and 110 nm size because it is an intensity-based technique [27] and a broad peak with high PDI can appear. While to confirm the actual size, number-based technique such as TEM is needed [28]. Therefore, DLS data do not corroborate with TEM images. Since, hydrodynamic size is usually greater than the actual size [22,29]. 

TEM and SEM analysis revealed spherical shape of NPs with an average size of 64 nm and 73 nm respectively. Spherical shape is an indication of the presence of large sur-face area for interactions with siRNA moieties. The most crucial characteristic for the in vivo integrity and biological fate of NPs is their particle size [30]. The development of car-riers that are the right size is crucial for the field of delivery of various moieties into the cell. Particle size <100 nm indicates that these NPs could easily penetrate the cell to show their effect [31]. 

Zeta potential of LNPs was observed to be +29 mV. The electric potential of NPs is re-ferred as zeta potential, and it is influenced by both the composition of the particles and the dispersing media. The colloidal suspension system is thought to be stable when it contains NPs with zeta potentials greater than +30 mV or less than -30 mV, which inhibits NP aggregation [32]. Since the zeta potential obtained in the study is near +30 mV, we can conclude that the LNPs are stable. Moreover, positive potential indicates that our NPs could bind to negatively charged nucleic acid [Figure 1].

### 3.2. Biocompatibility of the LNP

**Figure 2:**
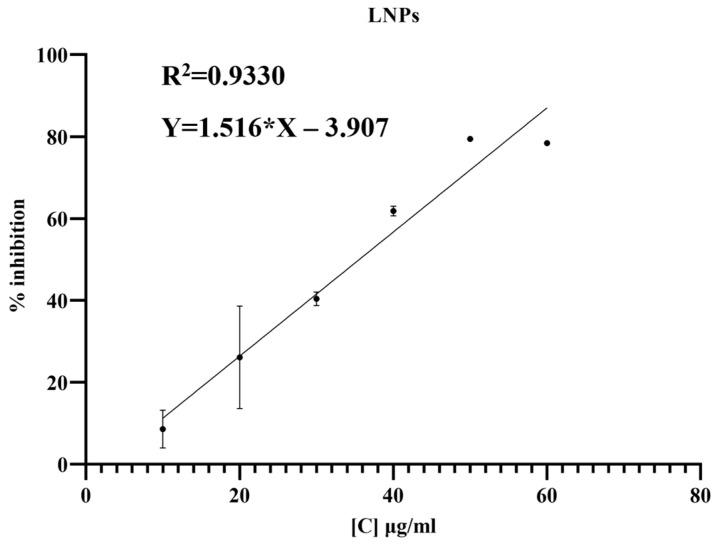
MTT assay to calculate IC_50_ for synthesized LNP.

The cell metabolic activity by MTT analysis revealed that synthesized LNP were not cytotoxic to the RAW264.7 even at concentrations as high as 30 µg/mL. This strengthened the idea that they can be used as siRNA delivery agent without causing any adverse effect [Figure 2].

### 3.3. Binding Efficiency of siRNA with Lipid Nanoparticle (LNPs)

**Figure 3:**
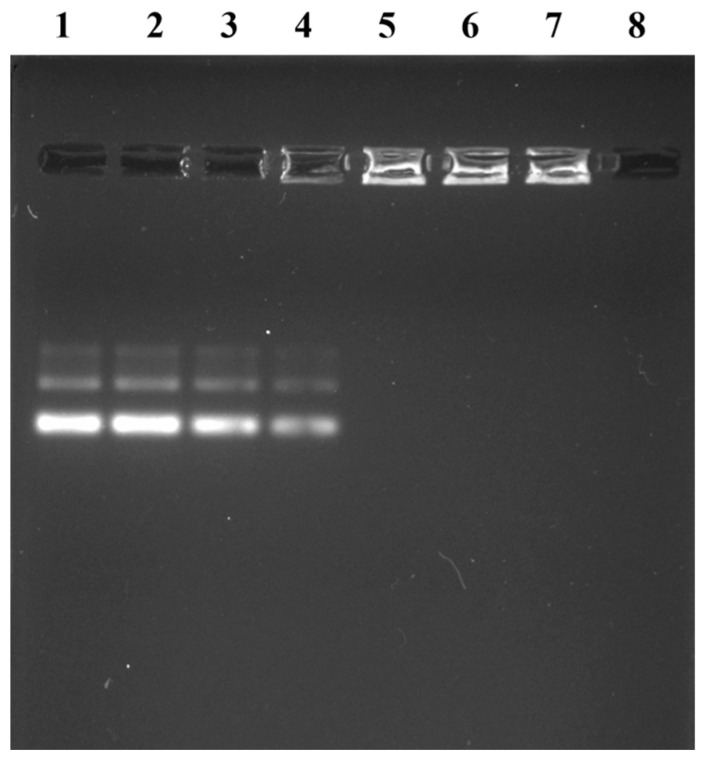
Nanoparticles Binding with siRNA. Nanoparticles 1 µg to 7 µg (left to right) while RNA is same (10 µg) in each of the well. Well 8 contains only nanoparticles.

Optimizing the delivery parameters and siRNA transfer into cells depend on nanoparticles’ ability to form complexes with siRNA ex vivo. To evaluate how well the nanoparticles and siRNA interacted, the gel retardation experiment was used. After 10 µg of siRNA was incubated with 1–7 μg of NPs and were run for agarose gel electrophoresis. Because of the bigger size, siRNA complexes with the NPs and remain in the loading holes in the agarose gel and could not be moved by the electric field. The detected bands indicated the presence of free siRNA, and siRNA-LNPs complexes showed concentration-dependent gel retardation. The best ratio of siRNA:LNPs was found to be 5:2 where no bands were detected in the gel but in the wells and beyond this ratio, we observed no bands of free siRNA in the gel. It indicates that to load 10 µg siRNA completely 4 µg LNPs is required [Figure 3].

### 3.4. Detection of Gene-Silencing through qRT-PCR

**Figure 4:**
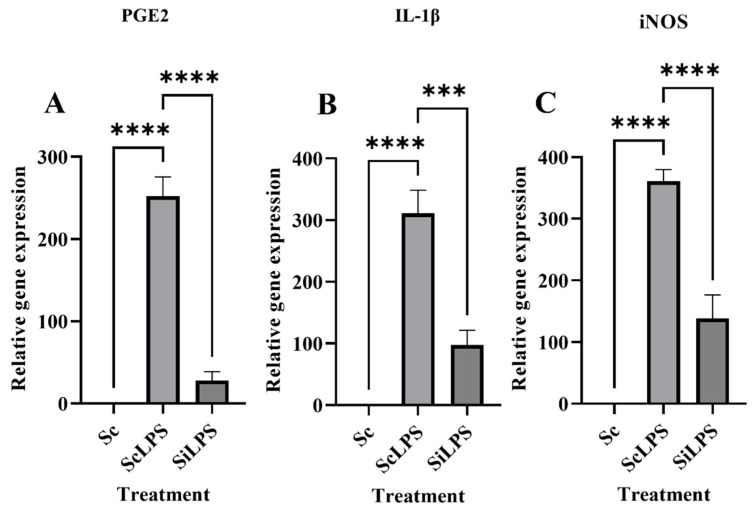
Quantitative analysis of gene expression of (**A**) *PGE2*, (**B**) *iNOS* and (**C**) *IL-1β* after Lipid nanoparticles mediated delivery of anti-PGE2 siRNA. Where, Sc = scrambled + LNPs, scLPS = scrambled + LPS, siLPS = siRNA + LPS. Here **** indicates the level of significance between Sc, ScLPS and SiLPS, whereas *** indicates level of significance between ScLPS and SiLPS.

Gene-silencing efficiency of nanoparticle-delivered siRNA is observed by delivering, 4 μg of nanoparticles and 10 μg of siRNA mixture in RAW264.7 (siRNA + LPS + LNPs) in a 6 well plate. In another set of cells, mixture of 4 μg of LNP and 10 μg of scrambled RNA (scrambledRNA + LPS + LNPs) were added. The cells were incubated for 24 h. RNA was extracted as well as studied further using quantitative real-time PCR. As shown in Figure 4, the mRNA level of PGE2 was significantly reduced by LNP-delivered anti-PGE2-siRNA in LPS stimulated cells (siLPS + LNPs) as compared to the LNP-delivered scrambled in LPS stimulated cells (scLPS + LNPs). This silencing of PGE2 gene expression indicated possibility of augmentation of macrophage induced inflammation. This was further confirmed by assessing the expression of pro-inflammatory markers iNOS and IL-1β [Figure 4].

### 3.5. Western Blot

**Figure 5:**
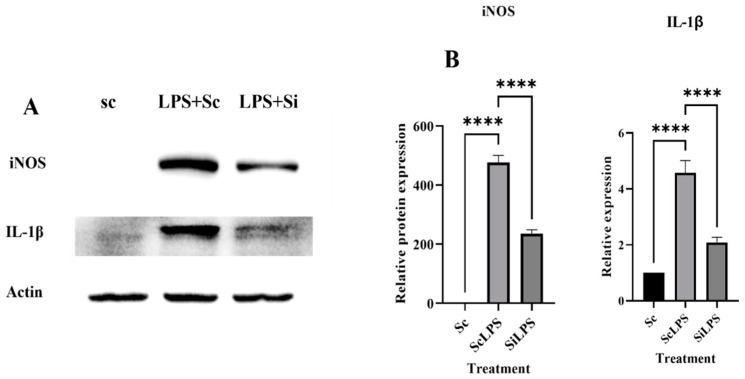
(**A**) iNOS and IL-1β expression determined by western blot analysis (**B**) Represent densitometric analysis done by ImageJ software (1.53r). Here **** indicates the level of significance between Sc, ScLPS and SiLPS.

Western blot analysis revealed a significant decrease in the expression of iNOS in LPS stimulated cells (siLPS) when anti-PGE-2-siRNA was delivered via LNPs to them as compared to the control i.e., scrambled RNA delivered cells (scLPS + LNPs). This indicates that inhibition of PGE2 expression is inhibiting iNOS expression affecting M1 polarization [Figure 5].

## 4. Discussion

PGE2 is derived from arachidonic acid and is a major physiologically active lipid. PGE2 is abundantly produced during acute inflammation and edema and is blocked by NSAIDs, can resolve the inflammation [33,34]. But these drugs can cause serious effects on gastrointestinal and cardiovascular system [35]. To downregulate PGE2 here we designed or synthesized lipid nanocarriers using DSPC:Cholestrol:PEI system. Synthesis was con-firmed by DLS, Zeta analyzer SEM and TEM analysis. 2:5 ratio of NPs and siRNA was a suitable ratio identified by gel retardation assay. The procedure successfully deliver siR-NA to the macrophage cells RAW264.7 to silence PGE2 gene and further inflammatory pathways and macrophage polarization markers were assessed. PGE2 is involved in macrophage metabolism during inflammation and these cells are crucial for immunolog-ic response, pathogen response, tissue repair, regeneration [36], and metabolism thus maintaining tissue homeostasis [33,37,38,39]. Similarly, M1 macrophages also release the potent pro-inflammatory cytokine IL-1β. Due to the activation of the NF-kB and MAPK cascades, very high quantities of IL-1β are present in M1 polarized macrophages, while M2 polarized macrophages do not contain any IL-1β protein [40,41]. Downregulation of IL-1β expression is an indication towards anti-inflammatory M2 macrophage polarization and reduction in M1 polarization [42]. IL-1β can be produced and induce inflammation in response to the Toll-like receptor 4-ligand lipopolysaccharide (LPS). Recently, in murine bone marrow-derived macrophages, it has been reported that PGE2 can induce an inflammatory response by stimulating the production of 1L-1β through cAMP/protein kinase signaling and inhibiting TNF-alpha [43]. PGE2 was also reported to boost the ability of LPS to induce pro-1L-1β expression. Similarly, suppression of PGE2 can affect the LPS-induced 1L-1β expression adversely through a positive feedback loop. Similar observations are also made in our study. We observed that nanoparticle-mediated silencing of PGE2 significantly decreased LPS-induced 1L-1β expression and inflammatory response.

The study indicates that expression of IL-1β also decreased in siLPS + LNPs as compared to scLPS + LNPs. These results affirmed that siRNA mediated silencing of PGE-2 can decrease proinflammatory polarization of macrophages via affecting iNOS and IL-1β expressions.

Limitation of this study, PEI-based liposomes were synthesized along with DSPC which is known for its lower toxicity. However, in the current scenario toxicity of nanoparticles was only checked against the cell line and further work needs to be done on the animal model in order to acquire the complete information related to their siRNA leakage, accumulation in the reticuloendothelial system, release from the body and stability in the circulatory system.

## 5. Conclusions

In the quest of gene delivery system, Lipid nanoparticles gene delivery system might be very prominent and safe. In the present study, PEI based lipid nanoparticles were synthesized with positive zeta potential. Successful PGE2 siRNA delivery using DSPC:Cholestrol:PEI nanoconjugates showed less toxicity to RAW264.7 macrophages and higher gene delivery efficiency. Inhibition of PGE2 significantly reduce inflammatory markers in LPS treated macrophages. From this study we can conclude that DSPC:Cholestrol:PEI nanoconjugates can be served as good biocompatible and efficient nanocarrier system for the inhibition of selected genes in the cellular system.

## Data Availability

All data have been included within the manuscript.
